# Identification of Ribonuclease Inhibitors for the Control of Pathogenic Bacteria

**DOI:** 10.3390/ijms25158048

**Published:** 2024-07-24

**Authors:** Rute G. Matos, Katie J. Simmons, Colin W. G. Fishwick, Kenneth J. McDowall, Cecília M. Arraiano

**Affiliations:** 1 Instituto de Tecnologia Química e Biológica António Xavier, Universidade Nova de Lisboa, Avenida da República, 2780-157 Oeiras, Portugal; 2 Astbury Centre for Structural Molecular Biology, School of Biomedical Sciences, Faculty of Biological Sciences, University of Leeds, Leeds LS2 9JT, UK; k.j.simmons@leeds.ac.uk; 3 Astbury Centre for Structural Molecular Biology, School of Chemistry, Faculty of Engineering and Physical Sciences, University of Leeds, Leeds LS2 9JT, UK; c.w.g.fishwick@leeds.ac.uk; 4 Astbury Centre for Structural Molecular Biology, School of Molecular and Cellular Biology, Faculty of Biological Sciences, University of Leeds, Leeds LS2 9JT, UK; k.j.mcdowall@leeds.ac.uk

**Keywords:** RNase II, RNase R, PNPase, antimicrobials, virtual high-throughput screening (vHTS)

## Abstract

Bacteria are known to be constantly adapting to become resistant to antibiotics. Currently, efficient antibacterial compounds are still available; however, it is only a matter of time until these compounds also become inefficient. Ribonucleases are the enzymes responsible for the maturation and degradation of RNA molecules, and many of them are essential for microbial survival. Members of the PNPase and RNase II families of exoribonucleases have been implicated in virulence in many pathogens and, as such, are valid targets for the development of new antibacterials. In this paper, we describe the use of virtual high-throughput screening (vHTS) to identify chemical compounds predicted to bind to the active sites within the known structures of RNase II and PNPase from *Escherichia coli*. The subsequent in vitro screening identified compounds that inhibited the activity of these exoribonucleases, with some also affecting cell viability, thereby providing proof of principle for utilizing the known structures of these enzymes in the pursuit of new antibacterials.

## 1. Introduction

Pathogenic bacteria are responsible for many diseases of medical relevance today, such as food poisoning, meningitis, and pneumonia. Currently, most of the antimicrobial compounds in clinical use are derivatives of molecules discovered over 50 years ago and are increasingly becoming ineffective in the treatment of infections due to the emergence of antibiotic resistance [1]. These antibiotics mainly target essential prokaryotic pathways involved in cell wall synthesis, protein translation, folate metabolism, RNA transcription, or DNA replication, with any off-target effects on the human cells being manageable [1].

Bacterial RNA metabolism offers a panoply of possible antimicrobial targets, since the molecular machinery involved has some differences between prokaryotes and eukaryotes; however, little work has been conducted for designing new drugs that target RNA metabolism [2]. Ribonucleases (RNases) are crucial players, since they modulate the processing, degradation, and quality control of RNAs. Moreover, it has been reported that they are involved in virulence processes in several pathogenic organisms (reviewed in [3]). For this reason, RNases can be considered as targets to develop novel therapeutic drugs. It was already demonstrated the use of small molecules that target an endoribonuclease in prokaryotes [4,5]. It was first shown that some compounds were able to specifically bind and inhibit *E. coli* and *Mycobacterium tuberculosis* RNase E and also RNase G, a paralogue of RNase E [4]. Later, three novel inhibitors design against RNase E were shown to be active against RNase E from *E. coli*, *Francisella tularensis*, and *Acinetobacter baumannii* [5]. These findings can be exploited for future drug discovery.

RNases can be divided into endoribonucleases, which cleave the RNA molecules internally, and exoribonucleases, which degrade the RNA by removing terminal nucleotides from the extremities of the RNA molecules. In this work, we used the major exoribonucleases involved in RNA degradation (PNPase, RNase II, and RNase R) as possible targets for the development of new antimicrobial compounds.

PNPase is considered a multifunctional protein: it is a phosphorolytic enzyme that processively degrades RNA from the 3′ end [6]. PNPase can act alone or in association with other proteins, such as the ones that form the complex termed the degradosome [7]. In *E. coli*, PNPase is not essential, unless either RNase II or RNase R are inactive [8]; however, it has been implicated in the establishment of virulence in several pathogens, such as *Salmonella* spp., *Campylobacter jejuni*, *E. coli* O157:H7, and *Yersinia* spp. [9,10,11,12]. PNPase is composed by three monomers linked in a ring-like shape [13]. Each monomer has two N-terminal RNase PH domains (PH1 and PH2), connected by an α-helical domain, which associate to form the central channel where catalysis occurs. At the C-terminus of the monomer, the S1 and KH domains are important for RNA binding and the stabilization of the trimer [13,14,15].

RNase II and RNase R are members of the RNB family of exoribonucleases. Proteins from this family are hydrolytic and progressively degrade RNA in the 3′ to the 5′ direction. These proteins play an important role in the cell. They were shown to be involved in stress responses as well as RNA and protein quality control [16]. Proteins from this family were shown to be important for virulence in several pathogenic organisms, such as *C. jejuni*, *Helicobacter pylori*, and *Streptococcus pneumonia* [3]. Prokaryotic members of the RNB family of enzymes have, at the N-terminal region, two cold-shock domains (CSD1 and CSD2), which are important for RNA binding, followed by the RNase II catalytic domain (RNB) and a S1 domain at the C-terminus, also involved in RNA binding [17]. As members of the same family, RNase II and RNase R share this domain organization; however, RNase R has an extra helix–turn–helix region at the N-terminus and a lysine-rich region after the S1 domain [18,19]. The structure of both proteins has been previously determined and shows that both share a similar overall structure, although they have different mechanisms of action [17,20].

The increasing prevalence of multidrug-resistant bacteria has become a major threat for global health. In recent years, the idea of reducing bacterial virulence has emerged as a way of neutralizing pathogens. As such, the use of compounds that target microbial virulence factors could be important in the effort against the growing threat of antimicrobial resistance. Such compounds are thought to apply less selective pressure, thus decreasing the probability of developing resistance compared to the conventional strategies. Moreover, the combination of different anti-virulence compounds with distinctive targets could be used to enhance the antimicrobial effect [21,22]. It was already demonstrated that the exoribonucleases RNase II, RNase R, and PNPase are important factors for virulence in several pathogenic bacteria [3]. Indeed, these ribonucleases pose as interesting targets for the design of new anti-virulence compounds. In this work, we used virtual high-throughput screening (vHTS) [23,24] to screen chemical structures that were predicted to bind to the active center of *E. coli* exoribonucleases RNase II and PNPase. Crystal structures were available for these enzymes at the start of this study. The crystal structure of *E. coli* RNase R only became available whilst we were testing the efficacy of the compounds. Thus, the compounds predicted to bind to PNPase and RNase II were also tested against RNaseR. We identified some chemical compounds that inhibit the activity of the RNases under study, with some of them also affecting cell viability. This work thus provides a proof of concept for the use of important ribonucleases for the discovery of new molecules that can act as antimicrobials.

## 2. Results

### 2.1. Selection of Small Molecules Targeting E. coli RNase II and PNPase by vHTS

**Figure 1:**
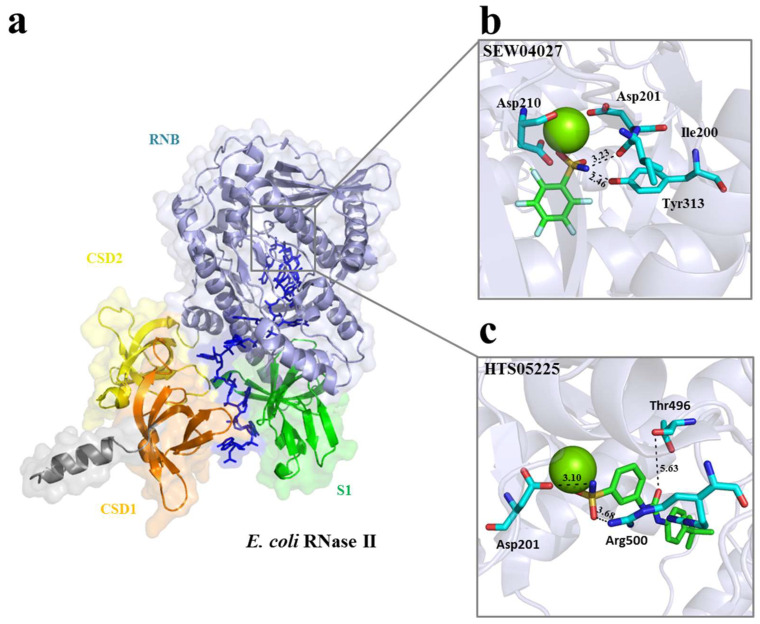
Structure of the *E. coli* RNase II catalytic domain and compound docking. (**a**) Overall structure of RNase II with bound RNA (blue). Orange, yellow, gray, and green coloring identifies the CSD1, CSD2, RNB, and S1 domains, respectively. (**b**) Predicted docking of SEW04027 in the RNase II catalytic site, where the key residues Ile200, D201, D210, and Y313 are depicted. The green sphere represents the Mg^2+^ ion. (**c**) Predicted docking of HTS05225 in the RNase II catalytic site, with the D201, T496, and R500 residues depicted. The green sphere denotes the Mg^2+^ ion. The images presented were generated using PyMOL [25].

**Figure 2:**
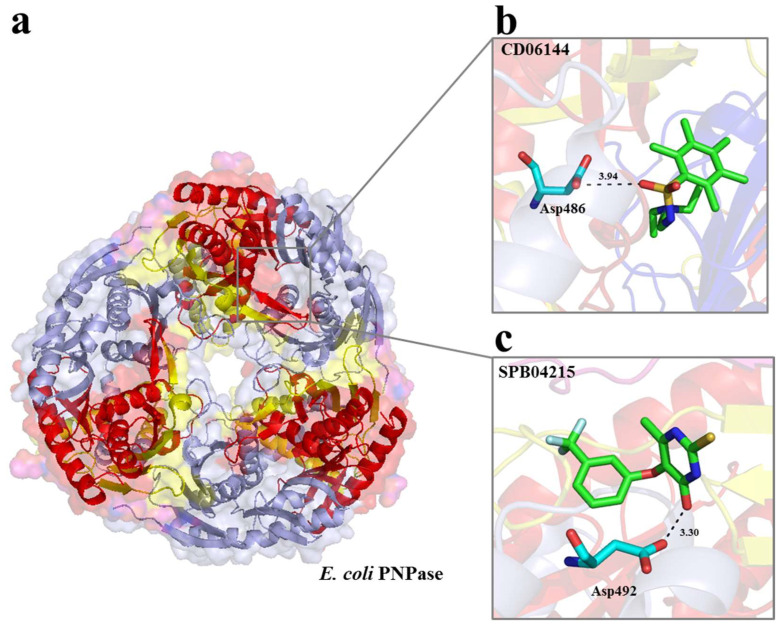
Structure of the *E. coli* PNPase core and compound docking. (**a**) Top view of the *E. coli* PNPase core trimer, showing the central channel. In each monomer, the RNase PH1 domain is colored in light blue, the α-helical region in yellow, and the RNase PH2 domain in red. RNA-binding domains KH and S1 are not shown. (**b**) Predicted docking of CD06144 in the PNPase catalytic site, where the key residue D486 is depicted. (**c**) Predicted docking of SPB04215 in the PNPase catalytic site, where the residue D492 is represented. The images presented were generated using PyMOL [25].

The vHTS was undertaken using the coordinates of the 2IX1 entry (2.74 Å resolution) for RNase II [17] and of the 3GCM entry (2.5 Å resolution) for PNPase [14]. Both of these structures were in a complex with RNA (Figure 1a and Figure 2a). For each, the segment of the RNA ligand within the active site was used to define a ‘cavity’ (i.e., a space available for ligand generation) using the CAnGAROO module of SPROUT. Then, the amino acid residues within an envelope that extended 10 Å in radius from the RNA were chosen to form the actual receptors for vHTS.

Subsequently, a total of 58,833 structures representing commercially available compounds in the Maybridge database were screened against the catalytic site of each of the enzymes using eHiTS. Compounds with an eHiTS score of ≤−4.0 were analyzed further using SPROUT. Those that also had a logp of ≤5.0 and SPROUT score values of ≤−4.9 were further analyzed to inspect the proposed binding modes as well as to assess those with drug-like properties. The final selection criterion was that the compounds should be predicted to form at least one specific hydrogen bond with either RNase II or PNPase. This was achieved using the ‘Explore’ function of the HIPPO module in SPROUT. This produced lists for RNase II and PNPase of 10 compounds against their active site (Table 1). The predicted binding mode of two small molecules to the active sites of RNase II (SEW04027 and HTS05225) and PNPase (CD06144 and SPB04215 for PNPase) is provided (Figure 1b,c and Figure 2b,c) and described further below.

### 2.2. RNase II Activity Is Affected In Vitro by Some Chemical Compounds

**Figure 3:**
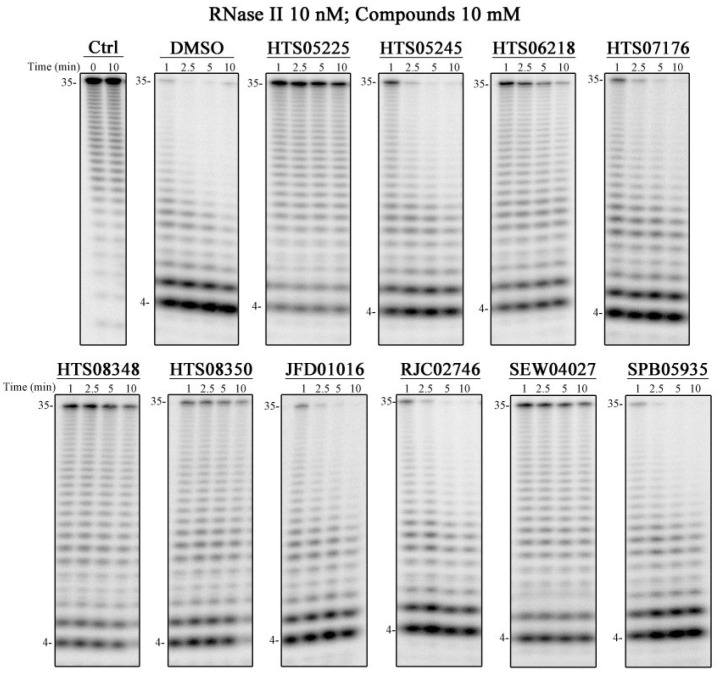
Exoribonucleolytic activity of RNase II in the presence of chemical compounds. A total of 10 nM of RNase II was incubated with 10 nM poly(A) and 10 mM of each compound at 37 °C for 10 min. Samples were taken during the reaction at the time points indicated in the figure. Ctrl, control without enzyme.

Each of the 10 compounds identified for RNase II were screened for inhibitory activity using a discontinuous assay and the conditions described in the Materials and Methods Section (Figure 3). Briefly, RNase II was incubated with an RNA substrate in the absence or presence of the chemical compound and the reactions analyzed using polyacrylamide gel electrophoresis, which allows the extent of RNA cleavage to be observed. Considering that all the chemical compounds were prepared in DMSO, and that this solvent could affect the activity assays, we ran a control reaction in which the protein was incubated with the same amount of DMSO but in the absence of any chemical compound (DMSO panel). We also added DMSO in the control reaction to confirm that the cleavage observed was not due to a contaminant but to the ribonuclease of interest.

Five of the compounds were able to significantly reduce the activity of RNase II: HTS05225, HTS06218, HTS08348, HTS08350, and SEW04027 (Figure 3). From these compounds, HTS05225 was the most efficient in inhibiting RNase II activity in vitro. Following our vHTS and the selection of compounds, the structure of *E. coli* RNase R became available [20]. It shares a common three-dimensional arrangement with RNase II with all the critical residues for exoribonucleolytic activity located in equivalent spatial positions [17,20]. Thus, we tested the five compounds that were able to inhibit RNase II activity against RNase R. As a negative control, we also tested these compounds against PNPase. HTS05225 and HTS08348, besides inhibiting RNase II activity, were found to inhibit RNase R, while the other three compounds (HTS06218, HTS08350, and SEW04027) did not have any effect (Supplementary Figure S1a). None of the compounds were able to inhibit PNPase (Supplementary Figure S1b). The lack of effects of these compounds on PNPase is consistent with their mode of action being specific for RNase II (and, in some cases, its close relative RNase R) rather than being more general (e.g., protein denaturation).

### 2.3. One Chemical Compound Inhibits RNase II Activity In Vivo

The five compounds found to inhibit RNase II in vitro were tested for their ability to inhibit the growth of cells dependent on the function of RNase II in the absence of PNPase (MG1655 Δ*pnp*) [8,26]. The double mutant is inviable since either functional PNPase or RNase II is required for cell viability [8,26]. As a control, the compounds were also tested against cells that are not dependent on functional RNase II due to the presence of PNPase (MG1655).

**Figure 4:**
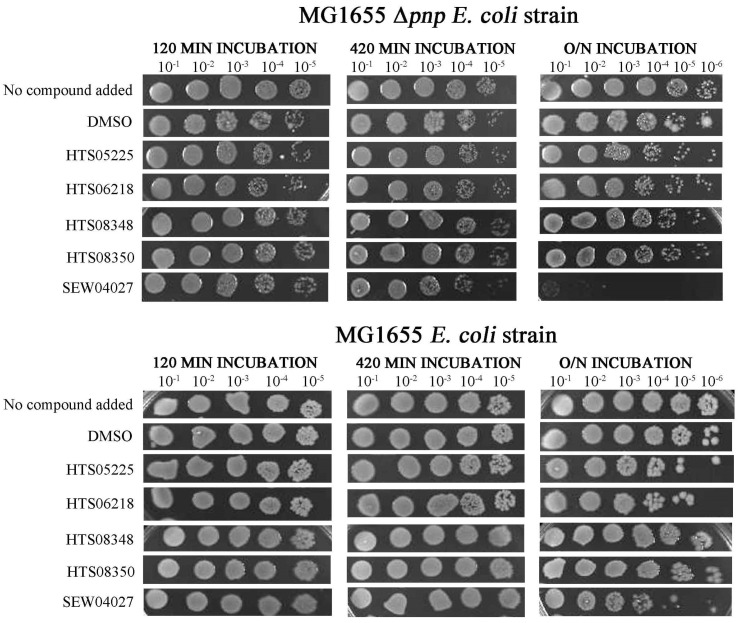
Representative viability analysis. MG1655 and MG1655 Δ*pnp* strains were incubated with 10 mM of the chemical compounds (LB media or DMSO were added in the control reactions). Samples were taken at the time points of 120, 420 min, and overnight.

Compared to the negative control (in the presence of DMSO), incubation with SEW04027 reduced cell viability following overnight incubation: distinct colonies were undetectable at the 10^−1^ dilution for SEW04027, but were detectable at the 10^−6^ dilution of the DMSO control (Figure 4, upper panels). Effects of SEW04027 were also detectable, although not as pronounced, following the 420 min: the 10^−5^ dilution for DMSO and the 10^−4^ dilution for SEW04027 produced similar number of colonies. The incubation of 120 min had no detectable effect. When SEW04027 was tested against cells whose growth was not dependent on functional RNase II (i.e., in the presence of functional PNPase), growth was readily detectable at the 10^−4^ dilution. In contrast, no growth was detected when cell viability was dependent on RNase II. Thus, SEW04027 affected cell growth via RNase II. However, the effects may also extend beyond RNase II: the growth of cells with functional PNPase was readily detectable at the 10^−5^ dilution when compared to growth in the presence of DMSO, which was still detectable at the 10^−6^ dilution.

The other four compounds, in cells whose growth was dependent on functional RNase II (MG1655 Δ*pnp*), when incubated for 120 or 420 min, did not affect cell growth. However, when incubation occurred for an overnight period, it is possible to see a decrease in cell viability, namely for the HTS05225 and HTS06218 compounds. However, the same effect was also observed in cells with functional PNPase (MG1655), suggesting an off-target effect.

### 2.4. Compounds That Affect PNPase Activity In Vitro

**Figure 5:**
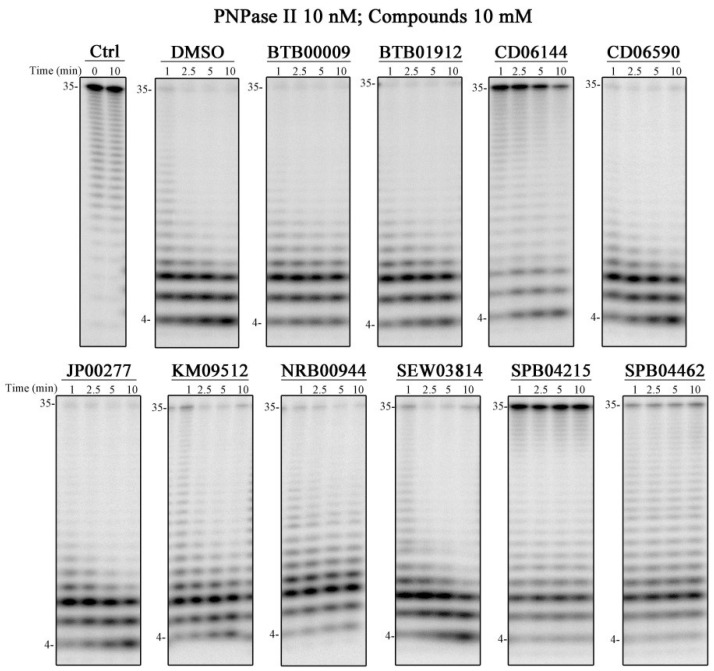
Exoribonucleolytic activity of PNPase in the presence of chemical compounds. A total of 10 nM of PNPase was incubated with 10 nM poly(A) and 10 mM each compound at 37 °C for 10 min. Samples were taken during the reaction at the time points indicated. Ctrl, control without enzyme.

Following the approach described above, each of the 10 compounds identified for PNPase were screened for inhibitory activity using a discontinuous assay and the conditions described in the Material and Methods Section. Three were found to inhibit PNPase activity: CD06144, SPB04215, and SPB04462 (Figure 5). From these, SPB04462 has a lower inhibitory effect when compared to the other two, considering that less RNA substrate is found at the end of the reaction (Figure 5). As a control, we also tested these compounds against RNase II and RNase R. CD06144 did not affect RNase II nor RNase R activity, suggesting that it is specific for PNPase (Supplementary Figure S2). However, the SPB04215 and SPB04462 compounds were also effective in inhibiting RNase R activity, with RNase II being also affected, but to a lesser extent (Supplementary Figure S2).

### 2.5. Compounds That Inhibit PNPase Reduce Viability of Wild-Type Cells

**Figure 6:**
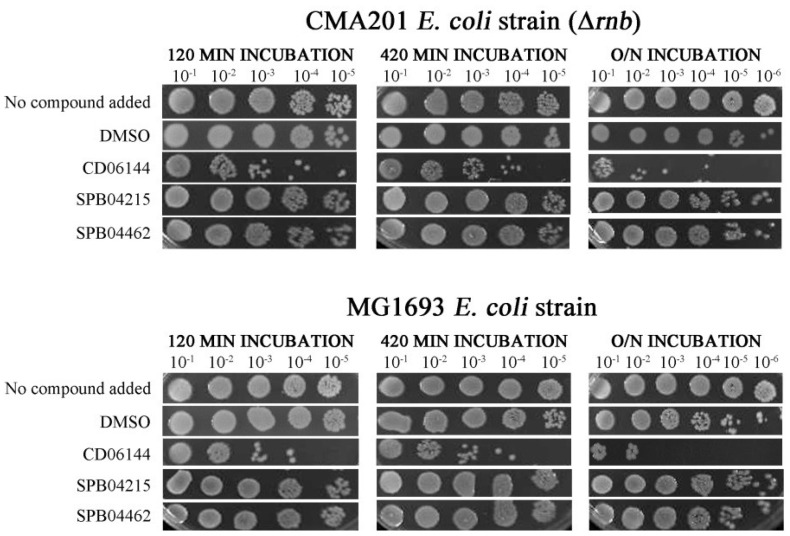
Representative viability analysis. CMA201 and MG1693 strains were incubated with 10 mM of the chemical compounds (LB media or DMSO were added in the control reactions). Samples were taken at the time points of 120, 420 min, and overnight.

The three compounds found to inhibit PNPase in vitro were also tested for their ability to inhibit the growth of CMA201 cells, which are dependent on PNPase function because of the absence of RNase II function. To provide a control, the compounds were also tested against MG1693 cells, which have RNase II function. As explained before, either PNPase or RNase II function is required for cell viability and the double mutant is not viable [8,26]. In the absence of a functional RNase II, the incubation of CMA201 cells with CD06144, but not the SPB04215 and SPB04462 compounds, resulted in a reduction in cell viability, when compared to the DMSO-only control. The reduction in cell viability was observed after incubation for 120 min (Figure 6, top panel) and was more pronounced after an overnight incubation, where viable cells were detected at the 10^−3^ dilution, while in the DMSO control viable cells were still detected at a 10^−6^ dilution (Figure 6, top panel). Similar results were obtained with the MG1693 cells (Figure 6, lower panel), indicating that CD06144 has cellular effects beyond PNPase.

## 3. Discussion

Bacteria are constantly adapting to antibiotics, and there is an increasing need to discover new molecules to fight against these pathogens. RNase II-like proteins and PNPase, as important virulence determinants, are interesting targets for the design of new anti-virulence compounds. In this paper, we show that the vHTS of compounds using the available crystal structures of these exoribonucleases has promise in identifying potential leads for the development of new antimicrobials. For both *E. coli* RNase II and PNPase, we identified compounds that had specificity in inhibiting their docking partner in vitro (see Figure 3 and Figure 5). When tested further in vivo, we identified SEW04027 for RNase II and CD06144 for PNPase that inhibited cell viability (see Figure 4 and Figure 6).

Compound SEW04027, which docks with RNase II, was specific for this target. As such, this could be a good antimicrobial to test its effectiveness against bacteria that lack PNPase and only have an RNase II homologue, such as *Mycoplasma genitalium* [27]. The RNase II homologue from *Mycoplasma* has 27% identity and 43% similarity to the *E. coli* RNase II [27], which strengthen the indication that our approach could be used for other bacteria. Our in vitro assays demonstrated that SEW04027 is specific for RNase II and is not able to inhibit RNase R activity. Although these two RNases present a similar structure, with most of the critical residues for catalysis located in comparable spatial positions [17,20], there are other factors that we should consider. For instance, the entry of the compound into the binding pocket my cause changes into the catalytic site [28], thus affecting its efficacy. To evaluate this, we could perform molecular dynamic simulations or solve the drug-bound structures. Nevertheless, SEW04027 seems to be a good scaffold to develop new versions more specific for RNase II-like proteins. We also identified two others compounds that inhibit RNase II activity in vitro, but that do not affect cell viability (HTS08348 and HTS08350). Biological systems are very complex and involve many more parameters than the ones that we can mimic in silico and in vitro. Although docking screenings are important to understand how chemical compounds can act on a certain target, they cannot simulate the cellular context, which will influence the final results [29]. Also, we have to take into consideration other mechanisms of intrinsic resistance, such as: (i) the limited uptake of the chemical compounds; (ii) the activity of efflux pumps that may pump out the compounds, thus reducing their intracellular concentration and effectiveness; and (iii) the inactivation of these chemicals before they can exert their effect [30]. Finally, when testing the compounds in vitro, they are confined to specific volumes, reaction conditions, and a known concentration of the compound and target protein. Inside the cells, these conditions will not be verified, thus explaining why certain compounds pass the in vitro test but fail in vivo.

The compounds HTS05225 and HTS06218, which inhibited RNase II activity in vitro, were able to affect cell viability beyond the targeting of RNase II. Also, CD06144 compound, which interacts with PNPase, was able to affect cellular viability. However, besides targeting PNPase, this compound appears to have off-target effects. When designing new chemical inhibitors, it is known that they can commonly exhibit off-target effects. Although it is impossible to screen all the interactions for every compound, in silico screenings combined with mechanistic analysis are being developed to assess off-targets [31], which may help progress in drug development. The rationale of our experimental design is to reduce bacterial viability by targeting important enzymes involved in RNA metabolism. As such, an off-target effect is not necessarily detrimental for our aim provided that it is not shared with mammalian cells. In fact, considering the inherent capacity that bacteria have to develop resistance to antibiotics, a compound with multiple cellular targets presents a highly advantageous strategy to combat the antibiotic resistance health crisis [32]. Moreover, there has been an increased interest in phenotypic drug discovery approaches due to its strong contribution for the discovery of first-in-class drugs [33]. In this work, by combining vHTS with in vitro and in vivo assays, we were able to provide the proof of concept for using the structures of RNase II and PNPase to find new anti-virulence compounds. As these proteins are important for the establishment of virulence in several human pathogens, our findings provide important insights for the development of anti-virulence drugs that could be synergistically used in combination with other antimicrobials. In this paper, we describe two chemicals that could be used as a scaffold to design improved versions using in silico approaches, thus improving their binding affinities and protein inhibition. It would also be interesting to expand our experimental work to other pathogenic bacterial species and find universal compounds targeting these conserved exoribonucleases that could be broadly used. Additionally, the new compounds may have other potential biotechnological applications, such as their use to study the effect of the activity of these proteins in organisms in which the construction of deletion mutants is not yet possible.

## 4. Materials and Methods

### 4.1. Materials

T4 polynucleotide kinase was purchased from Thermo Scientific (Waltham, MA, USA). Unlabeled Poly(A) substrate was synthesized by Thermolab (Waltham, MA, USA). The compounds listed in Table 1 were purchased from the Maybridge Chemical Company Ltd. (Altrincham, UK). The strains used in this study are described in Table 2. Luria broth (LB) and Luria agar (LA) were prepared as described previously [32]. When required, the media were supplemented with 0.4 mM thymine, 20 µg/mL tetracycline, 50 µg/mL kanamycin, and 40 µg/mL streptomycin (all from Sigma, St. Louis, MO, USA).

### 4.2. Molecular Modelling

To identify the compounds predicted to bind to the active sites of RNase II [17] and PNPase [14], we used the computational tools eHiTS and SPROUT. The eHiTS program, which is a molecular docking tool, has been described by Zsoldos et al. [38]. Briefly, eHiTS takes compounds from a library and calculates the optimal conformation that each of these ligands can adopt in a cavity of a protein target. The eHiTS approach breaks each ligand into rigid fragments and flexible connecting chains and docks each rigid fragment into every possible place in the cavity. A score is calculated for each structure based upon the geometries of the ligand and the complementarity of surface points on the receptor and ligand. Other factors are also used to calculate the final score, including steric clashes, depth of the cavity, solvation, intramolecular interactions in the ligand, and the conformational strain energy of the ligand. The score that is output is a logarithmic estimate of the binding efficiency for a particular ligand, such that the larger the negative score values, the greater the predicted affinity.

SPROUT [39] is a computer program that generates structures based upon a set of specified constraints. The approach firstly generates a skeleton based upon a set of primary constraints that includes the definition of the target site and must satisfy steric and geometric constraints. This is followed by the substitution of atoms in the skeleton to generate molecules with the required properties. The CAnGAROO module within SPROUT allows for the definition of potential binding pockets by detecting clefts, defined as a large inward facing area on the surface of the protein. The HIPPO module locates typical donor and acceptor atoms in the protein, intramolecular hydrogen bonds, hydrogen bonding atoms near to the surface of the receptor site, and hydrogen bonding regions, which are computed with tolerances. In a similar way to that for eHiTS, score values can also be obtained using SPROUT, where, again, the score that is output is a logarithmic estimate of the binding efficiency for a particular ligand, such that the larger the negative score values, the greater the predicted affinity.

### 4.3. Overexpression and Purification of E. coli RNase II, RNase R, and PNPase

The plasmids harboring the histidine-tagged proteins (Table 3) were introduced into *E. coli* BL21(DE3) by transformation to allow the expression of the recombinant proteins. For RNase II and PNPase, the cells were grown at 37 °C in 100 mL LB medium supplemented with 100 µg/mL ampicillin to an optical density measured at 600 nm (OD600) of 0.5, induced by the addition of 0.5 mM IPTG and grown at 37 °C for 2 h and 4 h, respectively. For RNase R, the cells were grown at 30 °C in 100 mL LB medium supplemented with 100 µg/mL ampicillin to an OD600 of 0.5, induced by the addition of 0.5 mM IPTG and grown at 16 °C for 16 h. The cells were pelleted by centrifugation and stored at −80 °C. Protein purification was performed by immobilized metal affinity chromatography using HiTrap Chelating HP columns (GE Healthcare) and the AKTA FPLC system (GE Healthcare), following the protocol described previously [40]. The purity of the proteins was verified by denaturing polyacrylamide gel electrophoresis and visualized by Coomassie blue staining. Proteins were quantified using the Bradford Method [41], and 50% (*v*/*v*) glycerol was added to the final fractions prior to storage at −20 °C.

### 4.4. Activity Assays

The activity assays were performed using the 35-nt Poly(A) oligomer as the substrate. Poly(A) was labelled at its 5′end with [γ^32^ATP] and T4 polynucleotide kinase. It was then purified using a G25 column (GE Healthcare) to remove the unincorporated nucleotides. The chemical compounds were dissolved in DMSO until a final concentration of 100 mM was obtained. The reaction buffer used contained 20 mM Tris–HCl (pH 8), 100 mM KCl, 1 mM MgCl_2_, and 1 mM DTT for RNase II and RNase R and 50 mM Tris–HCl (pH 8), 60 mM KCl, 1 mM MgCl_2_, 10 mM Na_2_HPO_4_ pH8, and 2 mM DTT for PNPase. The exoribonucleolytic reactions were carried out in a final volume of 20 µL containing 10 nM of Poly(A) and 10 mM of chemical compounds. The reactions were started by the addition of 10 nM of the enzyme and further incubated at 37 °C. As controls, the reaction was also performed in the absence of the enzyme (Ctrl) and in the absence of chemical compounds (DMSO). Samples were withdrawn at the time points indicated in the respective figures, and the reactions were stopped by the addition of formamide (95%) containing dye supplemented with 10 mM EDTA. The reaction products were resolved in a 8 M urea/20% polyacrylamide gel. Signals were visualized by PhosphorImaging and analyzed using the ImageQuant TL 8.1 software (Cytiva, Washington, DC, USA).

### 4.5. In Vivo Assays

Batch cultures from the strains MG1693, CMA150, HM104, MG1655, and Δ*pnp* were inoculated from overnight (16 h) in liquid cultures grown at 37 °C and 150 rpm (the inocula were diluted to an optical density of 0.08 measured at 600 nm). Cultures were grown aerobically at 37 °C and 150 rpm for 90 min; 20 µL of each chemical compound was added to 180 µL of each culture (to a final concentration of 10 mM of each compound) and further incubated at 37 °C and 150 rpm. As controls, DMSO and LB were also added to the cultures. At the time point indicated in the figures, 5 µL of culture was taken and processed by 10-fold serial dilutions in LB. Then, 10 µL of each dilution was plated on LA, and the plates were incubated at 37 °C overnight.

## Figures and Tables

**Table 1:** Chemical compounds from the Maybridge database used in this work.

Maybridge Code	eHiTS Score	Target	Name
BTB00009	−6.7	PNP	ethyl 4-(4-methylphenyl)-2-oxo-6-phenylcyclohex-3-ene-1-carboxylate
BTB01912	−6.8	PNP	2-[2-nitro-4-(trifluoromethyl)phenyl]-1,2,3,4-tetrahydroisoquinoline
CD06144	−7.4	PNP	N1-[3-(diethylamino)propyl]-2,3,4,5,6-pentamethylbenzene-1-sulfonamide
CD06590	−6.9	PNP	2,6-dimethyl-4-[(2,3,4,5,6-pentamethylphenyl)sulfonyl]morpholine
HTS05225	−7.1	RNB	3-({[4-(tert-butyl)anilino]carbonyl}amino)benzenesulfonamide
HTS05245	−6.9	RNB	N-[3-(aminosulfonyl)phenyl]-2,4-difluorobenzamide
HTS06218	−6.9	RNB	N-[3-(aminosulfonyl)phenyl]cyclohexanecarboxamide
HTS07176	−7.4	RNB	4-[(anilinocarbonyl)amino]benzenesulfonamide
HTS08348	−7	RNB	N-[4-(aminosulfonyl)phenyl]-4-ethylbenzamide
HTS08350	−6.9	RNB	N-[4-(aminosulfonyl)phenyl]-2,3-dimethylbenzamide
JFD01016	−7.0	RNB	ethyl 2-(3,4-dimethylanilino)-3,3,3-trifluoro-2-(propionylamino)propanoate
JP00277	−6.6	PNP	2,6-dimethyl-4-{[3-(trifluoromethyl)phenyl]sulfonyl}morpholine
KM09512	−7.0	PNP	2,8-di(trifluoromethyl)quinolin-4-ol
NRB00944	−7.4	PNP	2-[(4,5-diphenyl-1H-imidazol-2-yl)thio]acetic acid
RJC02746	−6.8	RNB	N1-[3-(2,3,4,5,6-pentafluorophenoxy)phenyl]acetamide
SEW03814	−7.1	PNP	4-oxo-4-[1-(trifluoromethyl)-2,3,4,9-tetrahydro-1H-beta-carbolin-2-yl]butanoic a
SEW04027	−6.9	RNB	2,3,4,5,6-pentafluorobenzenesulfonamide
SPB04215	−7.0	PNP	2-mercapto-6-methyl-5-[3-(trifluoromethyl)phenoxy]pyrimidin-4-ol
SPB04462	−7.3	PNP	N1-{4-hydroxy-6-methyl-5-[3-(trifluoromethyl)phenoxy]pyrimidin-2-yl}acetamide
SPB05935	−6.8	RNB	N1-[4-(trifluoromethyl)phenyl]-4-(trifluoromethyl)piperidine-1-carboxamide

**Table 2:** Strains used in this work.

*E. coli* Strain	Relevant Properties	Reference
Bl21(DE3)	F^−^ *r_B_*^−^ *m_B_*^−^ *gal ompT* [*int::P_lacUV5_ T7 gen1imm21 nin5*]	[34]
MG1693	thyA715 F^−^ *λ^−^rph-1*	[35]
CMA201	MG1693 Δ*rnb*-201*::tet*	[26]
MG1655	F^−^ *λ^−^rph-1*	[36]
MG1655_Δ*pnp*	MG1655 Δ*pnp::sr*	[37]

**Table 3:** Plasmids used in this work.

Plasmid	Characteristics	Reference
pFCT6.1	pET15b derivative expressing *E. coli* RNase II with a N-terminal Histidine tag	[42]
pABA–RNR	pET15b derivative expressing *E. coli* RNase R with a N-terminal Histidine tag	[40]
pABA–PNP	pET15b derivative expressing *E. coli* PNPase with a N-terminal Histidine tag	[40]

## Data Availability

The original contributions presented in the study are included in the article/Supplementary Materials, and further inquiries can be directed to the corresponding authors.

## Supplementary Materials

The following supporting information can be downloaded at: https://www.mdpi.com/article/10.3390/ijms25158048/s1.
