# Supplementary material for: Identification of Ribonuclease Inhibitors for the Control of Pathogenic Bacteria
Source: Int J Mol Sci. 2024 Jul 24;25(15):8048. doi: 10.3390/ijms25158048 (PMC11311990; doi:10.3390/ijms25158048)
Supplement: Supplementary file 1 [file ijms-25-08048-s001.zip › ijms-3099514-supplementary.pdf]

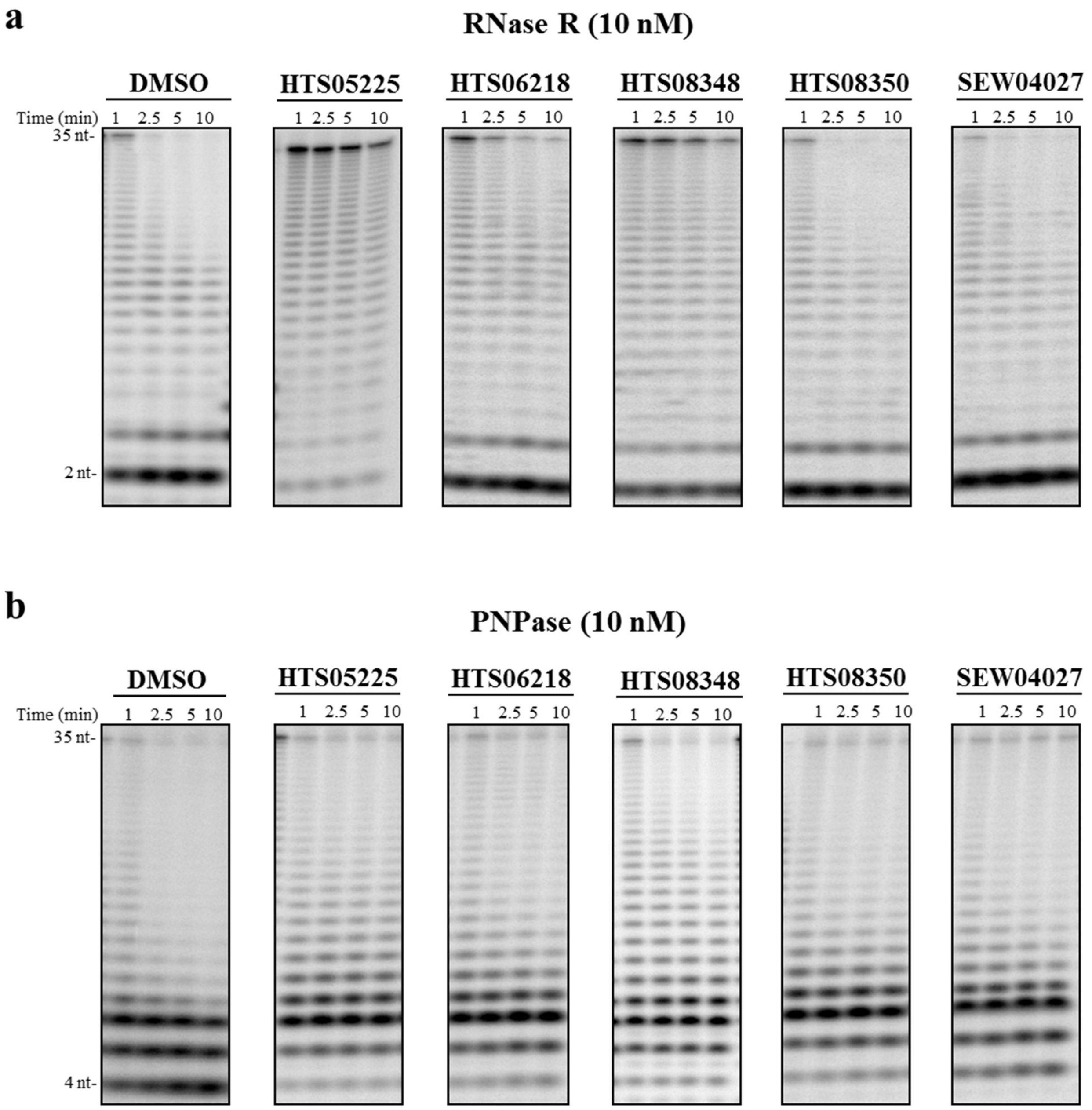

Supplementary Figure S1. Effect of the chemical compounds predicted to inhibit RNase II activity over other *E. coli* ribonucleases. a) 10 nM of RNase R were incubated with 10 nM poly(A) and 10 mM of each compound at 37°C for 10 min. **b)** 10 nM of PNPase were incubated with 10 nM poly(A) and 10 mM of each compound at 37°C for 10 min. Samples were taken during the reaction at the time points indicated in the figure. Ctrl, control without enzyme.

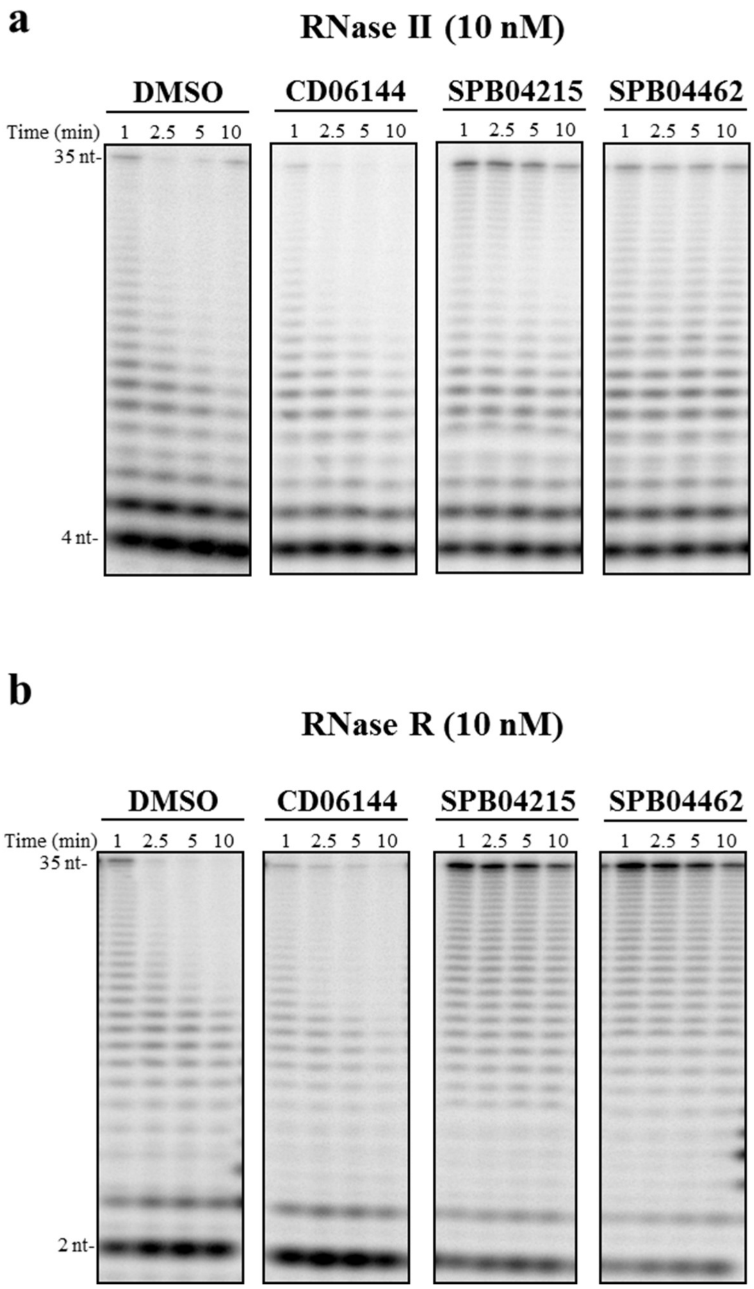

Supplementary Figure S2. Effect of the chemical compounds predicted to inhibit PNPase activity over other *E. coli* ribonucleases. a) 10 nM of RNase II were incubated with 10 nM poly(A) and 10 mM of each compound at 37°C for 10 min. **b)** 10 nM of RNase R were incubated with 10 nM poly(A) and 10 mM of each compound at 37°C for 10 min. Samples were taken during the reaction at the time points indicated in the figure. Ctrl, control without enzyme.
